# Stochastic hydration of a high-nitro­gen-content molecular compound recrystallized under pressure

**DOI:** 10.1107/S2052252521010381

**Published:** 2021-11-16

**Authors:** Anna Olejniczak, Anna Katrusiak, Marcin Podsiadło, Andrzej Katrusiak

**Affiliations:** a Faculty of Chemistry, Adam Mickiewicz University, Uniwersytetu Poznańskiego 8, Poznań 61-614, Poland; b Department of Organic Chemistry, Poznan University of Medical Sciences, Grunwaldzka 6, Poznań 60-780, Poland

**Keywords:** high-pressure crystallization, stochastic hydrates, group–subgroup relations

## Abstract

Partial hydration of organic compounds can be achieved by high-pressure crystallization. This has been demonstrated for the high-nitro­gen-content compound 6-chloro-1,2,3,4-tetrazolo[1,5-*b*]pyridazine (C_4_H_2_N_5_Cl), which becomes partly hydrated by isochoric crystallizations below 0.15 GPa. This hydrate, C_4_H_2_N_5_Cl·*x*H_2_O, is isostructural with the ambient-pressure phase α of C_4_H_2_N_5_Cl, but the crystal volume is somewhat larger than that of the anhydrate. At 0.20 GPa, the α-C_4_H_2_N_5_Cl anhydrate phase transforms abruptly into a new higher-symmetry phase, α′; the transformation is clearly visible due to a strong contraction of the crystals. The hydrate α-C_4_H_2_N_5_Cl·*x*H_2_O can also be isothermally compressed up to 0.30 GPa before transforming to the α′-C_4_H_2_N_5_Cl·*x*H_2_O phase. The isochoric recrystallization of C_4_H_2_N_5_Cl above 0.18 GPa yields a new anhydrous phase β, which, on releasing pressure, transforms back to the α phase below 0.15 GPa. The structural transition from the α to the β phase is destructive for the single crystal and involves a large volume drop and significant elongation of all the shortest intermolecular distances which are the CH⋯N and CH⋯Cl hydrogen bonds, as well as the N⋯N contacts. The α-to-α′ phase transition increases the crystal symmetry in the subgroup relation; however, there are no structural nor symmetry relations between phases α and β.

## Abstract 2

The ambient-pressure phase α of C_4_H_2_N_5_Cl transforms under 0.18 GPa to a higher-symmetry phase α′, but its high-pressure recrystallization below 0.20 GPa leads to a stochastic hydrate while above 0.20 GPa recrystallization leads to a new phase, β.

## 1. Introduction

**Figure 1:**
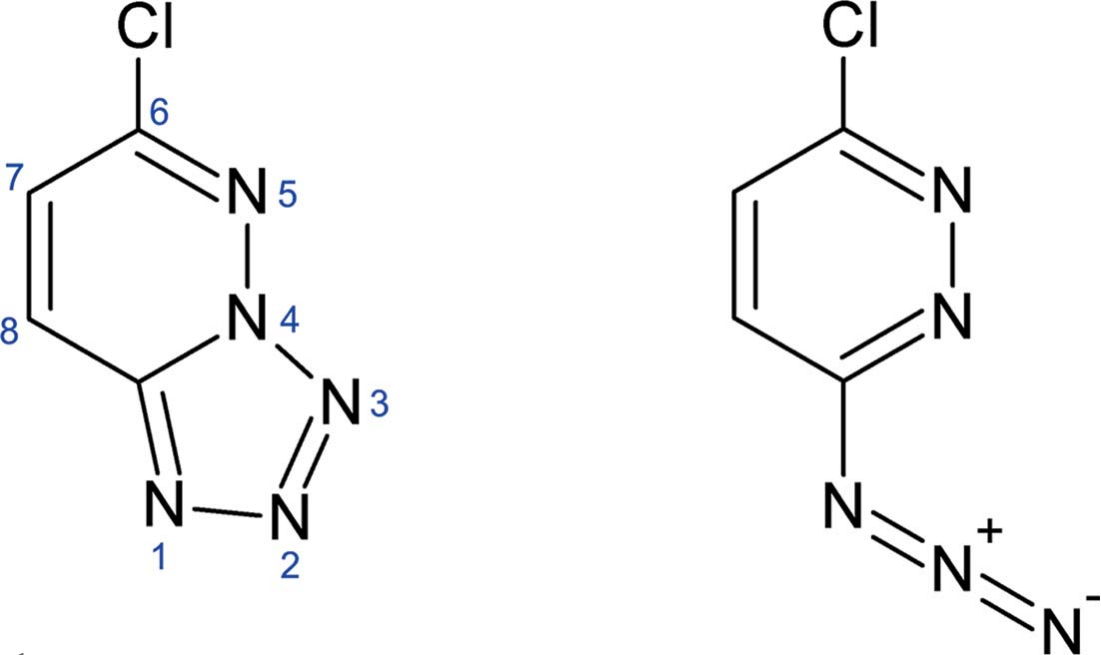
Structural formula of CTP and its azide tautomer.

High-nitro­gen organic compounds have relatively high density, but short intermolecular contacts are usually absent in their structures (Bernstein, 2002 ▸; Fabbiani & Pulham, 2006 ▸; Millar *et al.*, 2010 ▸; Zakharov & Boldyreva, 2019 ▸). The strong interdependence of the density and properties generally involves intermolecular interactions (Gao & Shreeve, 2011 ▸; Nair *et al.*, 2010 ▸) and thermodynamic conditions (Fabbiani & Pulham, 2006 ▸; Boldyreva, 2008 ▸, 2014 ▸; Resnati *et al.*, 2015 ▸). We report a pressure and temperature dependence of the crystal structure of the pyridazine-based compound 6-chloro-1,2,3,4-tetrazolo[1,5-*b*]pyridazine (C_4_H_2_N_5_Cl), hereafter CTP. It can transform between the azide and tetrazole forms in the gaseous and liquid states (Fig. 1 ▸). Azido-tetrazole tautomerism is common for many high-nitro­gen content compounds, widely applied as energetic materials and active pharmaceutical ingredients (Katrusiak *et al.*, 1996 ▸, 2005 ▸; Bałoniak & Katrusiak, 1994 ▸; Yang *et al.*, 2015 ▸; Olejniczak *et al.*, 2019 ▸). We determined the crystal structure of C_4_H_2_N_5_Cl under normal conditions in order to gain information about the tautomeric and molecular forms, and we noted relatively large voids, accommodating a probing sphere of 0.65 Å radius. Under ambient conditions the studied compound assumes the tetrazole form. We established that isothermal compression, isobaric cooling and high-pressure recrystallization result in new unexpected forms of CTP.

## 2. Methods

**Figure 2:**
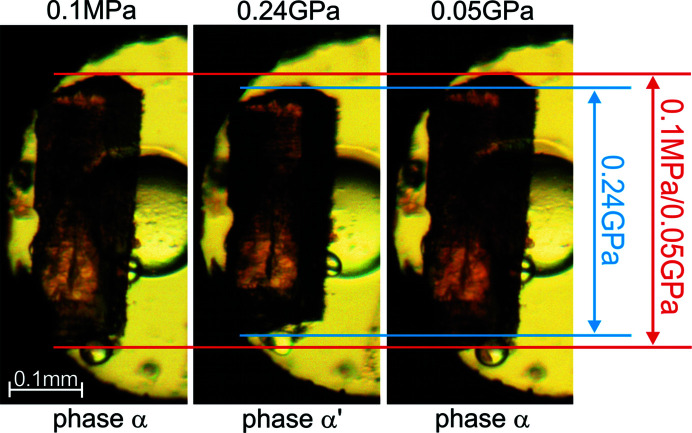
Single-crystal C_4_H_2_N_5_Cl in the α phase (0.1 MPa), compressed to the α′ phase (0.24 GPa) and decompressed to the α phase again (0.05 GPa). The vertical double arrows compare the initial and final vertical dimension of the crystal along its *x* direction in the α phase and corresponding *y* direction in the α′ phase (*cf.* Table 1 ▸).

The effect of high pressure on CTP was studied in a diamond anvil cell (DAC) (Merrill & Bassett, 1974 ▸) modified by mounting the anvils directly on steel disks with conical windows. Two procedures were applied (Fig. S1 of the supporting information): (i) isothermal compression (Figs. 2 ▸, S2 and S3) in Daphne oil and (ii) high-pressure recrystallizations performed from saturated solutions (Figs. 2 ▸ and S4). Method (i) resulted in monotonic compression of the ambient-pressure phase α up to 0.20 GPa, whereby the sample crystal visibly became shorter and transformed to a new phase α′ (Fig. 2 ▸). X-ray diffraction confirmed this to be a single-crystal-to-single-crystal phase transition of the subgroup–group symmetry relation, and clearly discontinuous in character.

**Figure 3:**
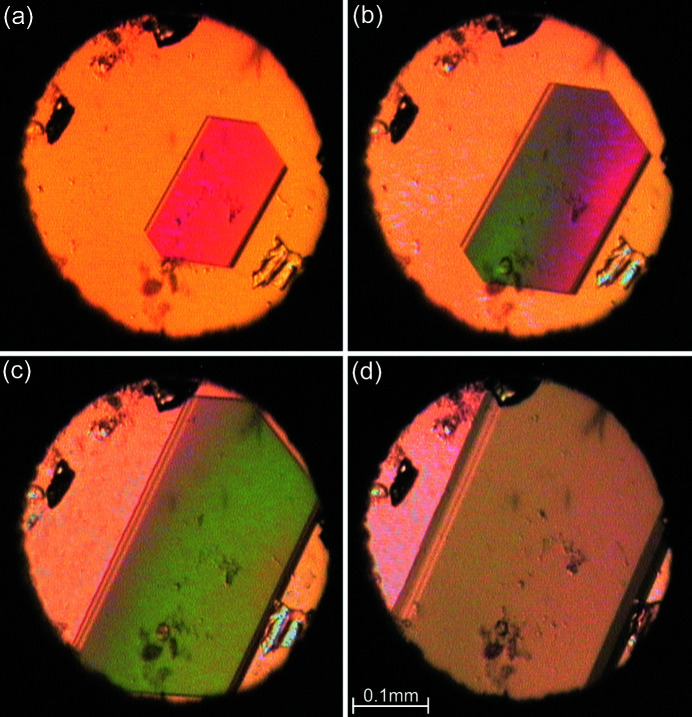
Stages of isochoric recrystallization of C_4_H_2_N_5_Cl in the β phase from acetone solution at 0.33 GPa: (*a*)–(*b*) a single crystal nucleated and growing in the DAC at 363 K; (*c*) the same crystal at 353 K and (*d*) at 296 K. Several ruby chips are mainly located close to the gasket edge.

For the high-pressure recrystallization, solvents were chosen according to their freezing pressure and the compound solubility. The highest solubility was found for water. The initial trials revealed that the concentration in the saturated solution is not sufficient for obtaining single crystals large enough for X-ray diffraction measurements. Therefore before loading the solution, some additional crystals were placed in the high-pressure chamber to increase the concentration at high temperature. After increasing the pressure to the required value, the DAC was heated until all seeds except one dissolved and a single crystal was grown by controlled slow cooling of the sample to room temperature (Fig. 3 ▸). High-pressure recrystallizations were performed from aqueous, methanol, ethanol and acetone solutions or from mixtures of them. Temperatures higher than 473 K caused the sample to decompose.

Pressure in the DAC chamber was calibrated by the ruby-fluorescence method (Mao *et al.*, 1985 ▸) with a photon control spectrometer affording an accuracy of 0.02 GPa; the calibration was performed before and after the diffraction measurements. The crystal sample in the DAC was centered on the diffractometer by the gasket shadowing method (Budzianowski & Katrusiak, 2004 ▸). For the low-temperature measurements an Oxford Cryosystems 700 Series attachment and SuperNova diffractometer using Cu *K*α radiation and a CCD plate Atlas detector was used. The high-pressure diffraction data were measured with a KUMA KM4-CCD diffractometer using Mo *K*α radiation and a CCD two-dimensional Eos detector. *CrysAlisPro* (171.40.67*a*; Rigaku Oxford Diffraction, 2019 ▸) was used for recording reflections and preliminary data reduction. Reflection intensities were corrected for the DAC absorption and sample shadowing by the gasket, the sample absorption and reflections overlapping with diamond reflections were eliminated. *OLEX2* (version 1.2, Dolomanov *et al.*, 2009 ▸), *SHELX-T* (Sheldrick, 2015*a* ▸) and *SHELX-L* (Sheldrick, 2015*b* ▸) were used to solve the structural models by direct methods, and then refine the models by full-matrix least-squares. Anisotropic temperature factors were applied for non-hydrogen atoms, but the isotropic thermal parameters were occasionally retained for the atoms with unreasonable anisotropic thermal ellipsoids. Hydrogen atoms were located from the molecular geometry, with the C—H distance equal to 0.93 Å and their *U*_iso_ factors constrained to 1.2 × *U*_eq_ of the carriers. The crystal data and refinement details are summarized in Tables 1 ▸ and S1–S3 of the supporting information; the experimental and structural details have been deposited in CIF format in the Cambridge Structural Database as supporting publications (CCDC deposition Nos. CCDC 2102408–2102436). Structural drawings were prepared using the *X-Seed* interface of *POV-Ray* (Barbour, 2001 ▸; Persistence of Vision Raytracer, 2004 ▸) and the program *Mercury* (Macrae *et al.*, 2020 ▸).

## 3. Results and discussion

High-pressure recrystallization revealed several crystalline forms of CTP. The β phase can easily be distinguished from phases α and α′ by the crystal morphology, but X-ray diffraction measurements were required for detecting the uptake of water molecules.

Crystals of the orthorhombic α phase were obtained from methanol solution under ambient conditions exclusively. The single-crystal sample isothermally compressed to 0.20 GPa displays an abrupt strong visible strain marking the transition to α′ (Figs. S2 and S3); subsequent X-ray measurements revealed the single crystal retains its high quality after the transformation is complete. This new high-pressure α′ phase remains orthorhombic, but its space group symmetry increases to *Pnma* (Table 1 ▸). On releasing pressure, α′ transforms back to α at 0.12 GPa. The α′ phase can be compressed to 0.6 GPa, when its transformation to the monoclinic β phase damages the single crystal. Single crystals of β were grown under isochoric conditions above 0.15 GPa. On releasing pressure, β transforms back to α at 0.15 GPa and the single crystal was pulverized again.

**Figure 4:**
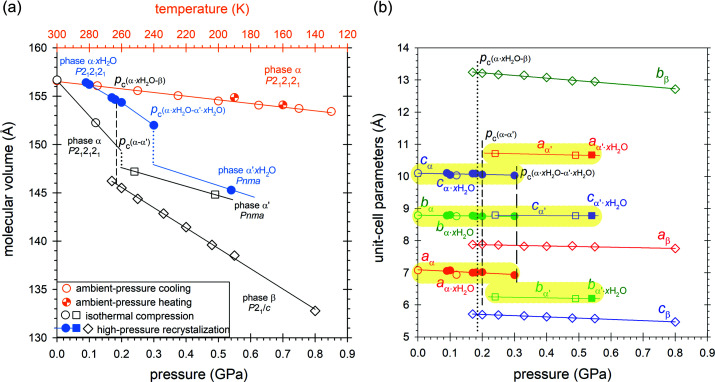
(*a*) Compression and thermal expansion of the molecular volume and (*b*) compression of the unit-cell dimensions of C_4_H_2_N_5_Cl (open symbols) and C_4_H_2_N_5_Cl·*x*H_2_O (full symbols). The compression line for the partial hydrate α′-C_4_H_2_N_5_Cl·*x*H_2_O (through one experimental point) is drawn parallel to that of the anhydrate α′-C_4_H_2_N_5_Cl as a guide for the eye only. All estimated standard deviations are smaller than the plotted symbols; the highlighted sections mark the corresponding dimensions of the α and α′ phases.

The high-pressure isochoric recrystallizations of CTP from aqueous solution up to 0.15 GPa yielded single crystals, which initially appeared to be identical to the α phase; however, their volume was markedly larger by about 3 Å^3^ per C_4_H_2_N_5_Cl molecule compared with that of the α phase grown at atmospheric pressure (Fig. 4 ▸). Moreover, the volume dependence on pressure displays clearly a convex shape, and the crystals could be compressed to 0.3 GPa before undergoing transformation to the α′ phase in an analogous way to that observed for the α phase. We concluded that the high-pressure recrystallization forces some water molecules into the crystal structure, so the inclusion compound C_4_H_2_N_5_Cl·*x*H_2_O is obtained, with H_2_O contents too small to be visible in our X-ray diffraction analysis. From isochoric recrystallizations above 0.16 GPa β was obtained, which is stable up to 0.80 GPa at least and on releasing pressure transforms back to α.

The low-temperature behavior of CTP crystals at atmospheric pressure was also studied by X-ray diffraction. Over the temperature range down to 130 K the crystal remained in the α phase and it contracted to about 98% of the volume at 296 K. Such a volume compression was achieved at 296 K under a pressure of 0.10 GPa (Fig. 4 ▸).

### 3.1. Symmetry relations

**Figure 5:**
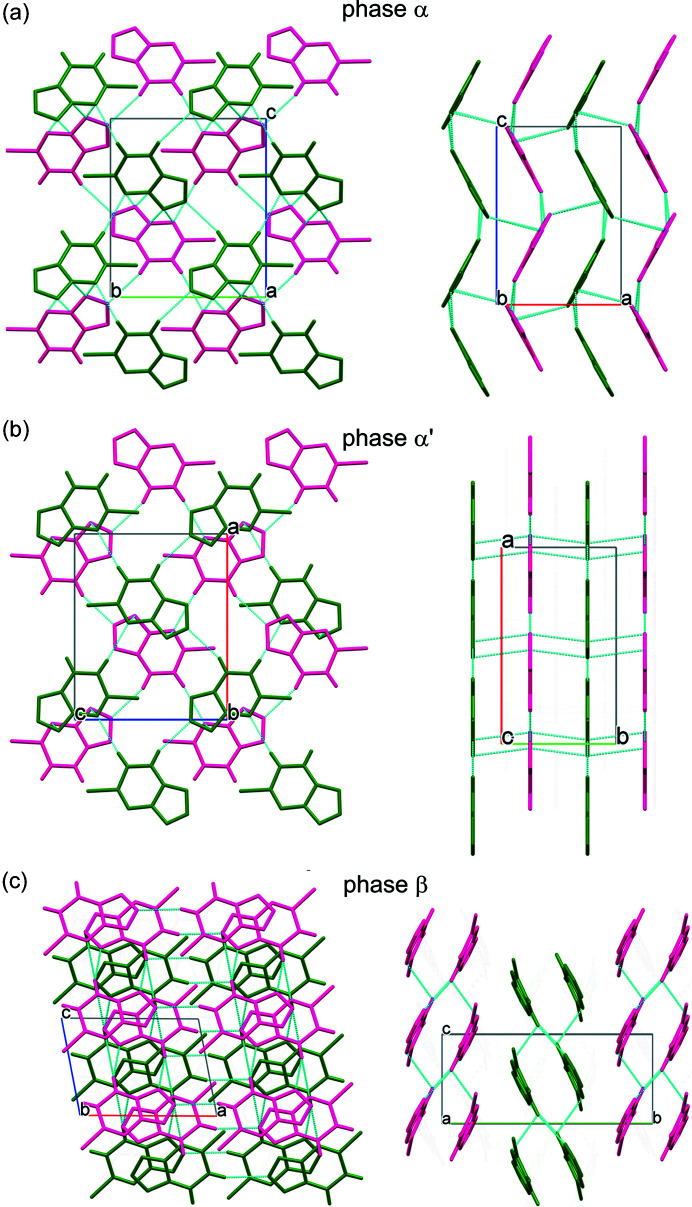
Molecular packing and the short contacts CH⋯N and N⋯N (dotted lines) in the C_4_H_2_N_5_Cl (*a*) α phase, (*b*) α′ phase and (*c*) β phase. Consecutive single layers (α and α′) and double layers (β) are marked in pink and green.

It is quite unusual and inconsistent with the rule of temperature and pressure inverse effects (*Tapie*) (Hazen & Finger, 1982 ▸; Cai & Katrusiak, 2014 ▸) that the space-group symmetry of the low-pressure phase α-CTP increases on transforming to the α′ phase, from *P*2_1_2_1_2_1_ to *Pnma*. *Tapie* states that the effects of increased pressure are usually the inverse of those of increased temperature (usually increasing volume and symmetry). Indeed, there are numerous examples of symmetry reduction in high-pressure phases (Olejniczak *et al.*, 2009 ▸, 2010 ▸; Svitlyk & Mezouar, 2021 ▸, Guńka *et al.*, 2021 ▸; Roszak & Katrusiak, 2021 ▸). The β phase is monoclinic (space group *P*2_1_/*c*), however its structure is very different from those of phases α and α′. The structure of β is built from double layers, which are absent in other phases (Figs. 5 ▸ and S5). The transition around 0.20 GPa between α and α′ can be observed visually, because the longest dimension of the crystal along the *x* direction of α is shortened by *ca* 10% at the transition to α′ (Fig. 4 ▸, Table 1 ▸). This visible strain precisely indicating the transition point on increasing and releasing the pressure was helpful for measuring the transition hysteresis (of about 0.08 GPa) as well as the higher transition pressure of the partial hydrate CTP·*x*H_2_O at 0.30 GPa.

Both phase transitions are accompanied by a volume reduction. The molecular volume of the β phase is over 10 Å^3^ smaller than that of the α phase (Fig. 4 ▸). At the α-to-α′ transition the molecular volume is reduced by about 3 Å^3^. The unit-cell volume of the isothermally compressed α phase is smaller compared with that of the sample recrystallized *in situ* under pressure. The *in situ* crystallized sample has the same symmetry and nearly identical structure and lattice as the α phase. The volume increase can be attributed to the presence of some small amount of water (Glasser, 2019 ▸) randomly distributed in the structure of α. We postulated that the presence of water in CTP·*x*H_2_O increases the crystal volume, reduces the compressibility of CTP·*x*H_2_O, and the transition to the α′ phase of CTP·*x*H_2_O occurs at pressures higher than that of the anhydrate (Fig. 4 ▸). Due to the absence of the water molecule electron-density peak in the Δ*F* maps, we were able to assess the value of *x* from only the volume increase of the *in situ* recrystallized crystals compared with those obtained under ambient conditions and compressed without recrystallization. This assessment has been based on the formula *x* = [*V*_m_(hydrate) − *V*_m_(anhydrate)]/*V*_w_, where *V*_w_ is the volume of one water molecule in a hydrate (Glasser, 2019 ▸). According to this assessment the water admixture coefficient *x* is about 0.14 for 0.10 GPa, 0.30 for 0.20 GPa and 0.08 for 0.49 GPa.

The structures of α and α′ are closely related (Fig. 5 ▸) and their lattice vectors are connected through the following matrix equations:

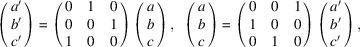

where primes refer to the α′ phase. The corrugated sheets of CH⋯N bonded molecular aggregates in α at the phase transition become perfectly planar in α′ (Fig. 5 ▸). Consequently, the lattice becomes elongated along the undulation of the sheets (unit-cell parameters *c*/*a*′), the other dimension along the sheets remains unchanged (*b*/*c*′) and the dimension between the sheets (*a*/*b*′) is shortened, as shown in the plot in Fig. 4 ▸(*b*).

**Figure 6:**
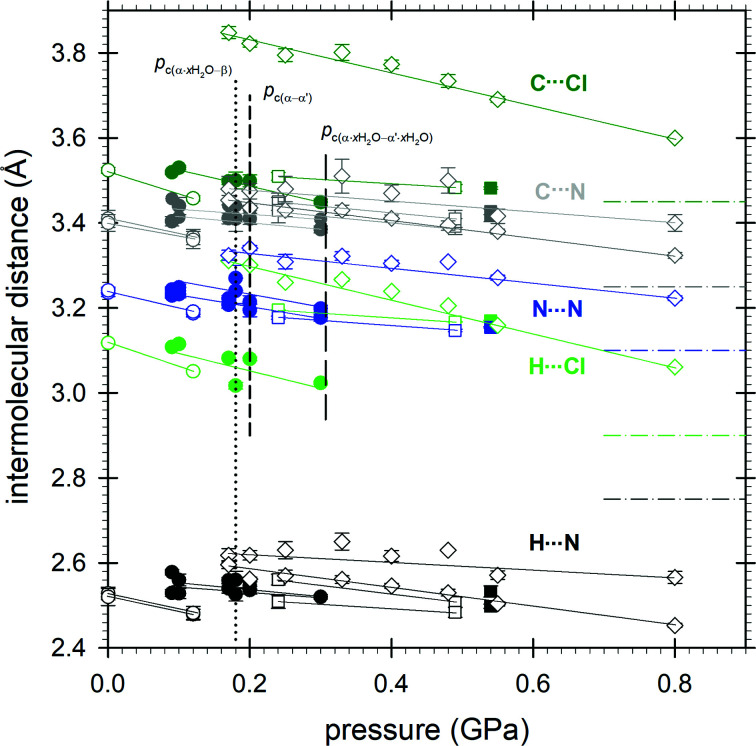
The shortest intermolecular distances plotted as a function of pressure in C_4_H_2_N_5_Cl (open symbols) and C_4_H_2_N_5_Cl·*x*H_2_O (full symbols): α phase (circles), α′ phase (squares), β phase (diamonds); distances H⋯N (black), C⋯N (gray), H⋯Cl (green), C⋯Cl (dark green), N⋯N (blue), sum of van der Waals radii (dash-dot lines).

In the tetrazole form the CTP molecules are rigid and planar under ambient conditions and these features are preserved in the high-pressure phases. All the structures are governed mainly by short CH⋯N bonds, while non contacts N⋯N, CH⋯Cl or Cl⋯Cl are shorter than the sum of van der Waals radii (Bondi, 1964 ▸) (Fig. 6 ▸). The arrangement of the molecules is clearly related between α and α′, but different from that in β (Fig. 5 ▸ and S10). All specific types of contacts in α become longer in β. Though the change in the CH⋯N distance is small (it is about 0.05 Å longer in phase β than it is in phase α), the elongation of distances N⋯N and CH⋯Cl is of the order of about 0.1 and 0.3 Å, respectively. This is due to the molecular arrangement becoming more optimized for denser packing rather than changes in the directional interactions under high pressure (Figs. S7 and S8).

The CH⋯N bonded corrugated sheets in α and the planar sheets in α′ are connected by N⋯N contacts between the sheets (Fig. 5 ▸). In β the two shortest CH⋯N bonds connect molecules into ribbons running along [100]; these ribbons are connected by other short CH⋯N bonds into double layers. There are short N⋯N contacts between these double layers. The patterns of molecules connected by the shortest CH⋯N contacts are rings that can be described as 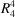 (17) in α and α′, and 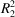 (8) and 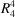 (12) in β (Fig. 5 ▸ and S9) according to graph notation (Etter *et al.*, 1990 ▸).

Some similarities can be observed between the structures of CTP phases and previously studied compounds 6-azido-1,2,4-triazolo[4,3-*b*]pyridazine (C_5_H_3_N_7_, ATriP) (Olejniczak *et al.*, 2019 ▸) and 6-azido-1,2,3,4-tetrazolo[1,5-*b*]pyridazine (C_4_H_2_N_8_, TAPYR) (Olejniczak *et al.*, 2020 ▸). Both CTP and TAPYR transform into new phases with considerably reduced volume. Moreover, the α-to-β transformation occurs only when the compounds are recrystallized *in situ* under high-pressure and high-temperature conditions. In TAPYR, the new β phase exists in the low-pressure range, then transforms to the ambient-condition α phase. In CTP at the low-pressure range the ambient-condition α phase is present, which further transforms to a new phase β. Unlike in TAPYR, where the new phase could be recovered after releasing the pressure, the β phase of CTP exists only under high-pressure conditions. In these three compounds, the molecules aggregate into sheets in phases α and α′ of CTP, in phase α of TAPYR, and in ATriP (planar in the α′ phase of CTP and in the α phase of TAPYR, while corrugated in the α phase of CTP and ATriP). The hydrogen-bond patterns are somewhat different (Fig. S9). In TAPYR the CH⋯N short contacts connect molecules into ribbons, which further extend through short N⋯N contacts into sheets. In CTP phases α and α′ and in ATriP the molecules aggregate into sheets only via CH⋯N bonds; however, in ATriP the N⋯N interactions are additionally present within the sheets. In all these structures N⋯N contacts are present between neighboring sheets, but they are longer than N⋯N contacts within the sheets.

The pressure-induced sorption of water molecules in small voids in the molecular crystal of CTP in some respect resembles the sorption in large pores of metal–organic frameworks (McKellar & Moggach, 2015 ▸), however access to the pores in CTP is hindered and requires dissolution.

## 4. Conclusions

With the exception of the three least-high-pressure phases of C_4_H_2_N_5_Cl we have revealed the different behavior of this compound compressed and recrystallized under high-pressure conditions. At ambient pressure and low temperature only the α phase was obtained. It transforms into the higher-symmetry α′ phase at 0.20 GPa and to phase β at about 0.70 GPa. The high-pressure recrystallization of C_4_H_2_N_5_Cl yields its stochastic hydrate α·*x*H_2_O when water is present in the solvent. This stochastic hydrate closely resembles the pure α phase, but its volume is somewhat larger; the volume–pressure dependence displays an unusual convex shape and the α·*x*H_2_O phase transforms into the α′·*x*H_2_O phase at a pressure about 0.1 GPa higher than the pure α-C_4_H_2_N_5_Cl phase. The presence of water can be also deducted from the voids present in the structure of phases α and α′, although their size is significantly smaller than that required for accommodating water molecules under ambient-pressure conditions.

## Supplementary Material

Crystal structure: contains datablock(s) C4H2N5Cl1@300K_phase_alpha, C4H2N5Cl1@275K_phase_alpha, C4H2N5Cl1@250K_phase_alpha, C4H2N5Cl1@225K_phase_alpha, C4H2N5Cl1@200K_phase_alpha, C4H2N5Cl1@190K_phase_alpha, C4H2N5Cl1@175K_phase_alpha, C4H2N5Cl1@160K_phase_alpha, C4H2N5Cl1@150K_phase_alpha, C4H2N5Cl1@130K_phase_alpha, C4H2N5Cl1@0_0001GPa_phase_alpha, C4H2N5Cl1@0_12GPa_phase_alpha, C4H2N5Cl1@0_24GPa_phase_alpha_prim, C4H2N5Cl1@0_49GPa_phase_alpha_prim, C4H2N5Cl1@0_09GPa_phase_alpha_xH2O, C4H2N5Cl1@0_10GPa_phase_alpha_xH2O, C4H2N5Cl1@0_17GPa_phase_alpha_xH2O, C4H2N5Cl1@0_18GPa_phase_alpha_xH2O, C4H2N5Cl1@0_20GPa_phase_alpha_xH2O, C4H2N5Cl1@0_30GPa_phase_alpha_xH2O, C4H2N5Cl1@0_54GPa_phase_alpha_prim_xH2O, C4H2N5Cl1@0_17GPa_phase_beta, C4H2N5Cl1@0_20GPa_phase_beta, C4H2N5Cl1@0_25GPa_phase_beta, C4H2N5Cl1@0_33GPa_phase_beta, C4H2N5Cl1@0_40GPa_phase_beta, C4H2N5Cl1@0_48GPa_phase_beta, C4H2N5Cl1@0_55GPa_phase_beta, C4H2N5Cl1@0_80GPa_phase_beta. DOI: 10.1107/S2052252521010381/lt5042sup1.cif

Supporting tables and figures. DOI: 10.1107/S2052252521010381/lt5042sup2.pdf

CCDC references: 2102408, 2102409, 2102410, 2102411, 2102412, 2102413, 2102414, 2102415, 2102416, 2102417, 2102418, 2102419, 2102420, 2102421, 2102422, 2102423, 2102424, 2102425, 2102426, 2102427, 2102428, 2102429, 2102430, 2102431, 2102432, 2102433, 2102434, 2102435, 2102436

## Figures and Tables

**Table 1:** Selected data of C_4_H_2_N_5_Cl recrystallized from different solutions (*cf.* Tables S1–S3 in the supporting information)

Formula	C_4_H_2_N_5_Cl	C_4_H_2_N_5_Cl·*x*H_2_O	C_4_H_2_N_5_Cl	C_4_H_2_N_5_Cl
*p* (GPa), *T* (K)	0.0001, 300.0 (1)	0.10 (2), 296 (2)	0.24 (2), 296 (2)	0.40 (2), 296 (2)
Phase	α	α	α′	β
Space group	*P*2_1_2_1_2_1_	*P*2_1_2_1_2_1_	*Pnma*	*P*2_1_/*c*
Unit-cell parameters				
*a* (Å)	7.0651 (2)	7.0733 (6)	10.697 (4)	7.824 (4)
*b* (Å)	8.7859 (2)	8.7965 (6)	6.2545 (7)	13.0789 (6)
*c* (Å)	10.0906 (2)	10.042 (8)	8.8012 (10)	5.6290 (4)
β (°)	–	–	–	100.793 (19)
Volume (Å^3^)	626.36 (3)	624.8 (5)	588.8 (3)	565.8 (3)
*Z*, *Z*′	4/1	4/1	4/0.5	4/1
*D*_calc_ (g cm^−3^)	1.650	1.654	1.755	1.826
Final *R*_1_/*wR*_2_ (I > 2σ_1_)	0.0340/0.0893	0.0339/0.0493	0.0249/0.0378	0.0619/0.1476
Solvent	–	MeOH:H_2_O (1:1)	–	Acetone
