# Supplementary material for: Stochastic hydration of a high-nitro­gen-content molecular compound recrystallized under pressure
Source: IUCrJ. 2021 Nov 16;9(Pt 1):49–54. doi: 10.1107/S2052252521010381 (PMC8733875; doi:10.1107/S2052252521010381)
Supplement: Supplementary file 2 [file m-09-00049-sup2.pdf]

IUCrJ

Volume 9 (2022)

Supporting information for article:

Stochastic hydration of a high-nitrogen-content molecular compound recrystallized under pressure

Anna Olejniczak, Anna Katrusiak, Marcin Podsiadło and Andrzej Katrusiak

Table S1 Selected crystal high-pressure data for $C_4H_2N_5Cl$ phases α and α·$x$$H_2O$.

	High-pressure recrystallization phase α·$x$$H_2O$						Isothermal compression phase α	
p (GPa)	0.09(2)	0.10(2)	0.17(2)	0.18(2)	0.20(2)	0.30(2)	0.0001	0.12(2)
T (K)	296(2)						296(2)	
Formula weight	155.56						155.56	
Wavelength (Å)	0.71073						0.71073	
Crystal system	Orthorhombic						Orthorhombic	
Space group	$P2_12_12_1$						$P2_12_12_1$	
Unit cell (Å,°) *a*	7.0542(4)	7.0733(6)	6.9987(3)	7.005(2)	7.0156(18)	6.9235(13)	7.0753(7)	6.9340(5)
b	8.7774(14)	8.7965(6)	8.7740(15)	8.7554(18)	8.7539(8)	8.7578(5)	8.7825(7)	8.7568(6)
c	10.1041(5)	10.042(8)	10.0863(9)	10.086(2)	10.0529(13)	10.0265(11)	10.099(10)	10.029(7)
Volume (Å^3)	625.62(11)	624.8(5)	619.37(12)	618.6(3)	617.39(19)	607.95(14)	627.5(6)	609.0(4)
Z/Z'	4/1	4/1	4/1	4/1	4/1	4/1	4/1	4/1
d_{calc} (g/cm^3)	1.652	1.654	1.668	1.670	1.674	1.700	1.647	1.697
Absorption coeff. (mm^{-1})	0.526	0.527	0.532	0.532	0.533	0.542	0.525	0.541
F(000)	312	312	312	312	312	312	312	312
Crystal size (mm)	0.39/0.29/0.18	0.38/0.29/0.21	0.37/0.27/0.16	0.38/0.26/0.14	0.28/0.22/0.10	0.26/0.22/0.10	0.30/0.30/0.11	0.30/0.30/0.11
R_{int}	0.0303	0.0493	0.0316	0.3126	0.1883	0.0391	0.0403	0.0390
Min/max indicies *h*	-8/8	-8/9	-8/8	-7/7	-6/6	-6/6	-8/8	-8/8
k	-6/6	-11/10	-7/7	-9/9	-11/11	-11/11	-11/11	-11/10
l	-12/12	-3/3	-11/11	-12/12	-11/12	-11/11	-3/3	-4/4
Θ-range for data collection (°)	4.034 to 27.175	3.696 to 27.176	4.238 to 27.070	3.081 to 28.147	3.086 to 28.562	4.267 to 27.346	4.213 to 26.963	4.264 to 29.790
Goodness-of-fit on F^2	1.058	1.110	1.087	1.156	1.002	1.078	1.038	1.034
Data/restrains/param.	746/0/91	491/6/91	666/0/91	965/0/92	923/0/91	815/0/91	475/0/91	487/0/91
Final R_1/wR_2 (I>2σ_1)	0.0359/0.0735	0.0339/0.0493	0.0371/0.0812	0.1430/0.3439	0.0617/0.0957	0.0425/0.0741	0.0318/0.0518	0.0297/0.0516
R_1/wR_2 (all data)	0.0636/0.0835	0.0613/0.0555	0.0585/0.0904	0.2367/0.4295	0.1692/0.1228	0.0665/0.0811	0.0620/0.0610	0.0572/0.0586
Solvent	ethanol	water:methanol(1:1)	ethanol	water:methanol(1:1)	methanol	methanol	--	--

Table S2 Selected crystal high-pressure data for $C_4H_2N_5Cl$ phases α' and α'·xH_2O.

	Isothermal compression of non-recrystallized sample α’		Isothermal compression of recrystallized sample α’·xH_2O
p (GPa)	0.24(2)	0.49(2)	0.54(2)
T (K)	296(2)		296(2)
Formula weight	155.56		155.56
Wavelength (Å)	0.71073		0.71073
Crystal system	Orthorhombic		Orthorhombic
Space group	*Pnma*		*Pnma*
Unit cell (Å,°) *a*	10.697(4)	10.654(3)	10.668(2)
b	6.2545(7)	6.1954(6)	6.2041(7)
c	8.8012(10)	8.7823(8)	8.780(4)
Volume ($Å^3$)	588.8(3)	579.69(17)	581.1(3)
Z/Z’	4/1	4/1	4/1
d_{calc} (g/cm^3)	1.755	1.782	1.778
Absorption coeff. (mm^{-1})	0.559	0.568	0.567
F(000)	312	312	312
Crystal size (mm)	0.30/0.26/0.11	0.30/0.26/0.11	0.40/0.25//0.20
R_{int}	0.0450	0.0442	0.0418
Min/max indicies *h*	-4/4	-4/4	-11/12
k	-7/7	-7/7	-7/7
l	-11/10	-10/11	-6/6
Θ-range for data collection (°)	4.429 to 26.915	4.457 to 26.982	4.453 to 27.115
Goodness-of-fit on F^2	1.081	1.135	1.082
Data/restrains/param.	263/6/61	268/12/61	321/0/61
Final R_1/wR_2 ($I>2σ_1$)	0.0249/0.0378	0.0292/0.0565	0.0382/0.0817
R_1/wR_2 (all data)	0.0495/0.0431	0.0567/0.0659	0.0693/0.0940

Table S3 Selected crystal high-pressure data for $C_4H_2N_5Cl$ phase β.

p (GPa)	0.17(2)	0.20(2)	0.25(2)	0.33(2)	0.40(2)	0.48(2)	0.55(2)	0.80(2)
T (K)	296(2)							
Formula weight	155.56							
Wavelength (Å)	0.71073							
Crystal system	Monoclinic							
Space group	$P2_1/c$							
Unit cell (Å,°) *a*	7.878(12)	7.885(6)	7.864(4)	7.824(8)	7.824(4)	7.836(2)	7.808(6)	7.756(5)
b	13.2446(15)	13.2116(6)	13.1668(6)	13.1476(8)	13.0789(6)	12.9703(12)	12.9522(8)	12.7163(5)
c	5.7134(11)	5.6949(3)	5.6843(4)	5.6603(6)	5.6290(4)	5.5885(5)	5.5722(4)	5.4680(3)
β	101.14(6)	101.20(2)	101.159(16)	101.05(4)	100.793(19)	100.542(16)	100.54(2)	100.006(2)
Volume ($Å^3$)	584.9(9)	582.0(4)	577.5(3)	571.4(6)	565.8(3)	558.43(18)	554.0(4)	531.1(3)
Z/Z'	4/1	4/1	4/1	4/1	4/1	4/1	4/1	4/1
d_{calc} (g/cm^3)	1.766	1.775	1.789	1.808	1.826	1.850	1.865	1.945
Absorption coeff. (mm^{-1})	0.563	0.566	0.570	0.576	0.582	0.590	0.594	0.620
F(000)	312	312	312	312	312	312	312	312
Crystal size (mm)	0.39/0.28/0.16	0.28/0.26/0.23	0.34/0.28/0.27	0.38/0.28/0.28	0.38/0.28/0.27	0.34/0.30/0.26	0.35/0.25/0.14	0.24/0.22/0.22
R_{int}	0.0423	0.0222	0.0400	0.0742	0.0259	0.0415	0.0307	0.0308
Min/max indicies *h*	-4/3	-2/2	-3/3	-3/3	-4/4	-3/3	-2/2	-2/2
k	-16/17	-16/17	-16/16	-17/17	-16/16	-16/16	-15/16	-15/16
l	-7/7	-7/7	-6/7	-7/7	-7/7	-7/6	-7/7	-6/6
Θ-range for data collection (°)	3.947 to 27.420	3.960 to 27.359	3.968 to 27.071	3.099 to 27.918	4.001 to 27.280	4.028 to 27.198	4.039 to 27.242	4.110 to 27.948
Goodness-of-fit on F^2	1.129	1.100	1.158	1.067	1.080	1.043	1.098	1.130
Data/restrains/param.	424/0/91	361/0/91	379/0/91	366/0/41	433/0/41	359/0/41	367/0/91	355/0/41
Final R_1/wR_2 ($I>2\sigma_1$)	0.0349/0.0713	0.0207/0.0477	0.0340/0.0570	0.0598/0.1455	0.0619/0.1476	0.0544/0.0953	0.0222/0.0441	0.0538/0.1280
R_1/wR_2 (all data)	0.0768/0.0844	0.0288/0.0509	0.0777/0.0665	0.0996/0.1766	0.0760/0.1578	0.0932/0.1117	0.0337/0.0480	0.0607/0.1326
Solvent	water	water:methanol(1:1)	water	acetone	acetone	water	water:methanol(1:1)	methanol

Table S4 Selected crystal low-temperature data for phase α.

T (GPa)	300.0(1)	275.0(1)	250.0(1)	225.0(1)	200.0(1)	175.0(1)	150.0(1)	130.0(1)	160.0(1)	190.0(1)
Formula weight	155.56									
Wavelength (Å)	1.54184									
Crystal system	Orthorhombic									
Space group	$P2_12_12_1$									
Unit cell (Å,°) *a*	7.0651(2)	7.0604(2)	7.0533(2)	7.0476(2)	7.0422(2)	7.03420(10)	7.02910(10)	7.02450(10)	7.0336(2)	7.0473(2)
b	8.7859(2)	8.7800(2)	8.7742(2)	8.7682(2)	8.7617(2)	8.7569(2)	8.75390(10)	8.75170(10)	8.7575(2)	8.7636(2)
c	10.0906(2)	10.0712(2)	10.0552(2)	10.0363(2)	10.0158(2)	10.0049(2)	9.9932(2)	9.9817(2)	10.0065(2)	10.0299(2)
Volume (Å^3)	626.36(3)	624.32(2)	622.29(3)	620.19(3)	617.99(3)	616.28(2)	614.902(17)	613.638(17)	616.37(3)	619.44(3)
Z/Z'	4/1	4/1	4/1	4/1	4/1	4/1	4/1	4/1	4/1	4/1
d_{calc} (g/cm^3)	1.650	1.655	1.660	1.666	1.672	1.677	1.680	1.684	1.676	1.668
Absorption coeff. (mm^{-1})	4.768	4.783	4.799	4.815	4.832	4.846	4.857	4.867	4.845	4.821
F(000)	312	312	312	312	312	312	312	312	312	312
Crystal size (mm)	0.50/0.20/0.10	0.50/0.20/0.10	0.50/0.20/0.10	0.50/0.20/0.10	0.50/0.20/0.10	0.50/0.20/0.10	0.50/0.20/0.10	0.50/0.20/0.10	0.50/0.20/0.10	0.50/0.20/0.10
R_{int}	0.0180	0.0180	0.0172	0.0170	0.0169	0.0163	0.0162	0.0155	0.0158	0.0176
Min/max indicies *h*	-6/8	-6/8	-6/8	-6/8	-8/6	-8/6	-6/8	-6/8	-6/8	-8/6
k	-9/10	-10/9	-10/9	-9/10	-10/9	-10/9	-9/10	-9/10	-9/10	-10/9
l	-10/11	-11/10	-11/10	-11/10	-11/10	-10/11	-10/11	-10/11	-10/11	-10/11
Θ-range for data collection (°)	6.681 to 64.687	6.689 to 64.795	6.696 to 64.632	6.705 to 64.718	6.713 to 64.809	6.719to 64.892	6.723 to 64.822	6.728 to 64.438	6.718 to 64.888	6.708 to 64.768
Goodness-of-fit on F^2	1.098	1.056	1.084	1.115	1.072	1.056	1.066	1.074	1.067	1.086
Data/restrains/param.	1046/0/91	1044/0/91	1038/0/91	1034/0/91	1032/0/91	1029/0/91	1023/0/91	1019/0/91	1028/0/91	1032/0/91
Final R_1/wR_2 (I>2σ_1)	0.0340/0.0893	0.0330/0.0834	0.0325/0.0849	0.0320/0.0838	0.0304/0.0789	0.0302/0.0767	0.0305/0.0780	0.0292/0.0741	0.0308/0.0790	0.0344/0.0903
R_1/wR_2 (all data)	0.0360/0.0913	0.0346/0.0848	0.0338/0.0860	0.0330/0.0846	0.0314/0.0797	0.0309/0.0772	0.0309/0.0783	0.0295/0.0743	0.0313/0.0797	0.0354/0.0911

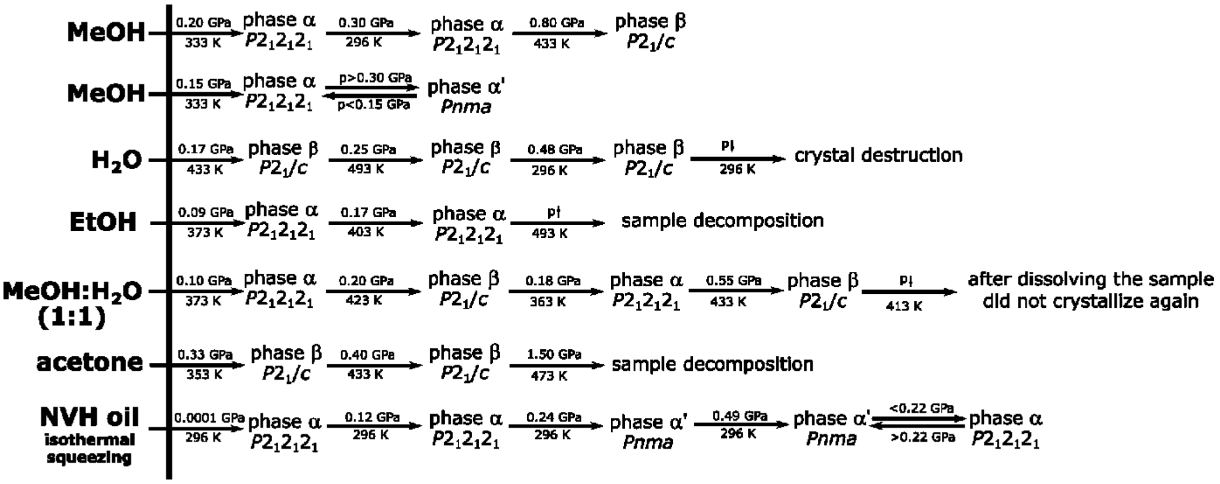

Figure S1 Conditions of formation of $C_4H_2N_5Cl_1$ polymorphs α, α' and β.

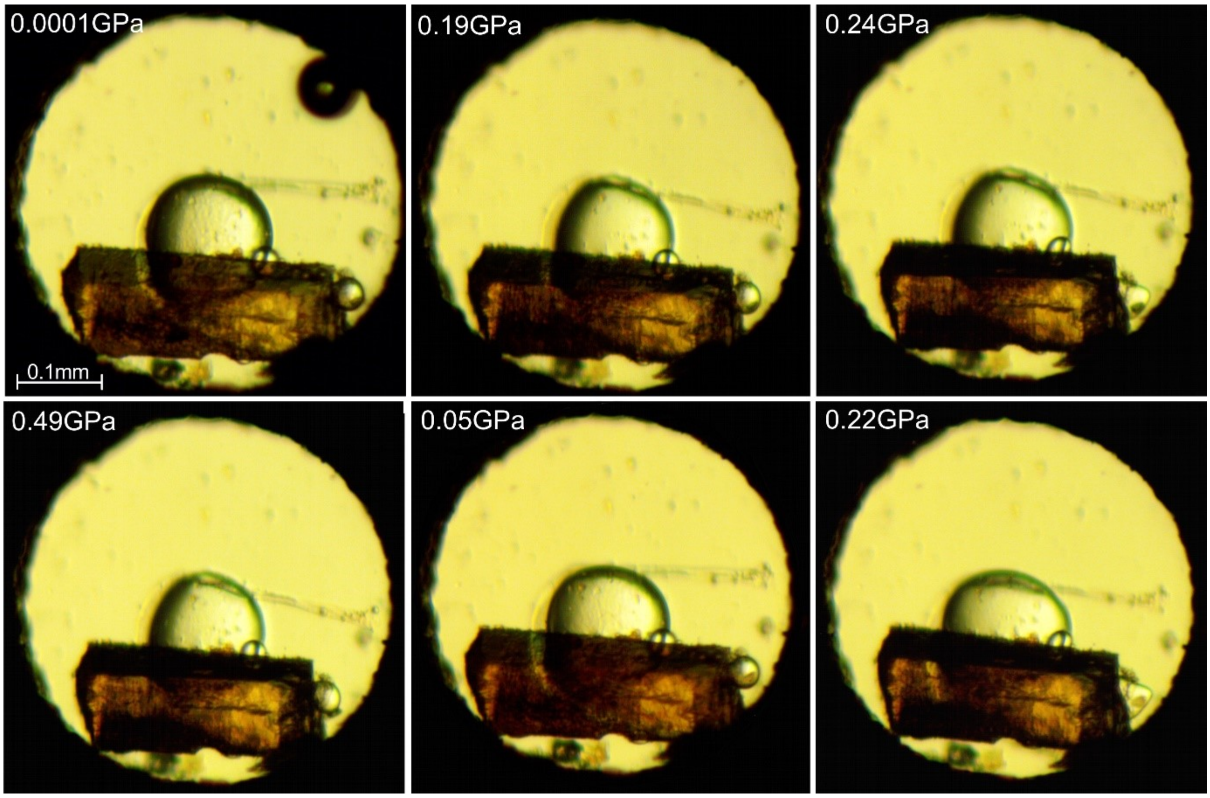

Figure S2 Stages of isothermal compression and decompression of $C_4H_2N_5Cl$.

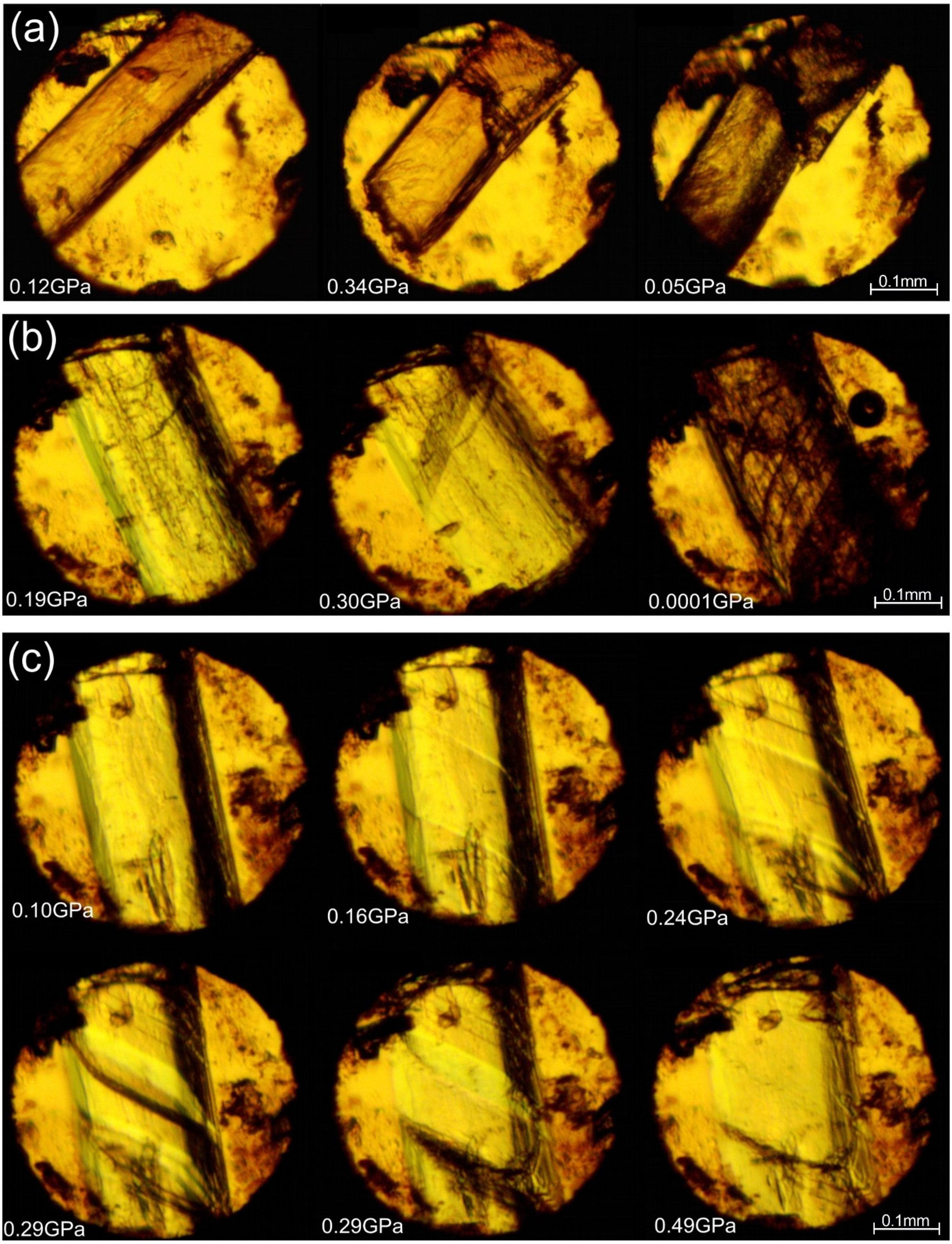

Figure S3 Isothermal compression of α·xH$_2$O obtained after recrystallization from aqueous solution (a-c) three different samples.

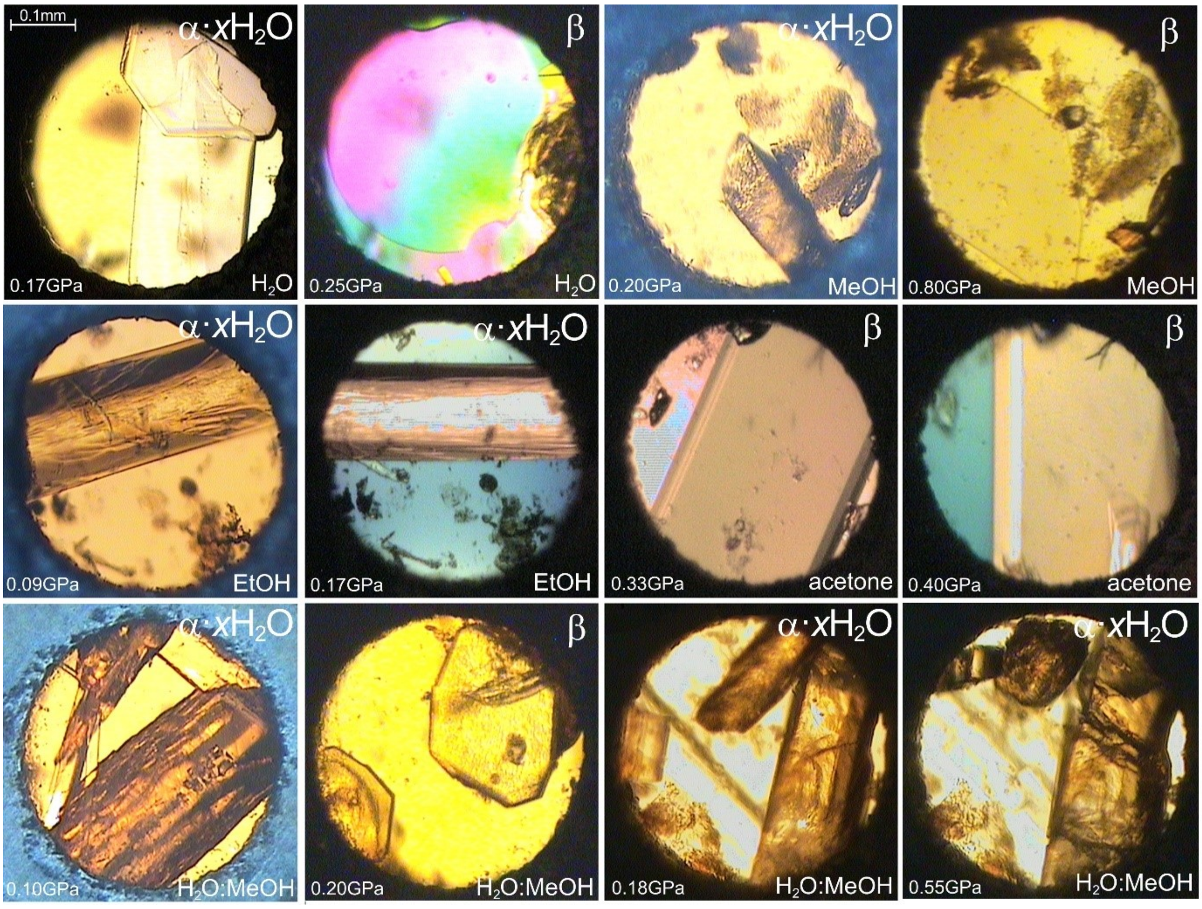

Figure S4 Single crystals of $C_4H_2N_5Cl$ at 296 K, obtained from different high-pressure recrystallizations.

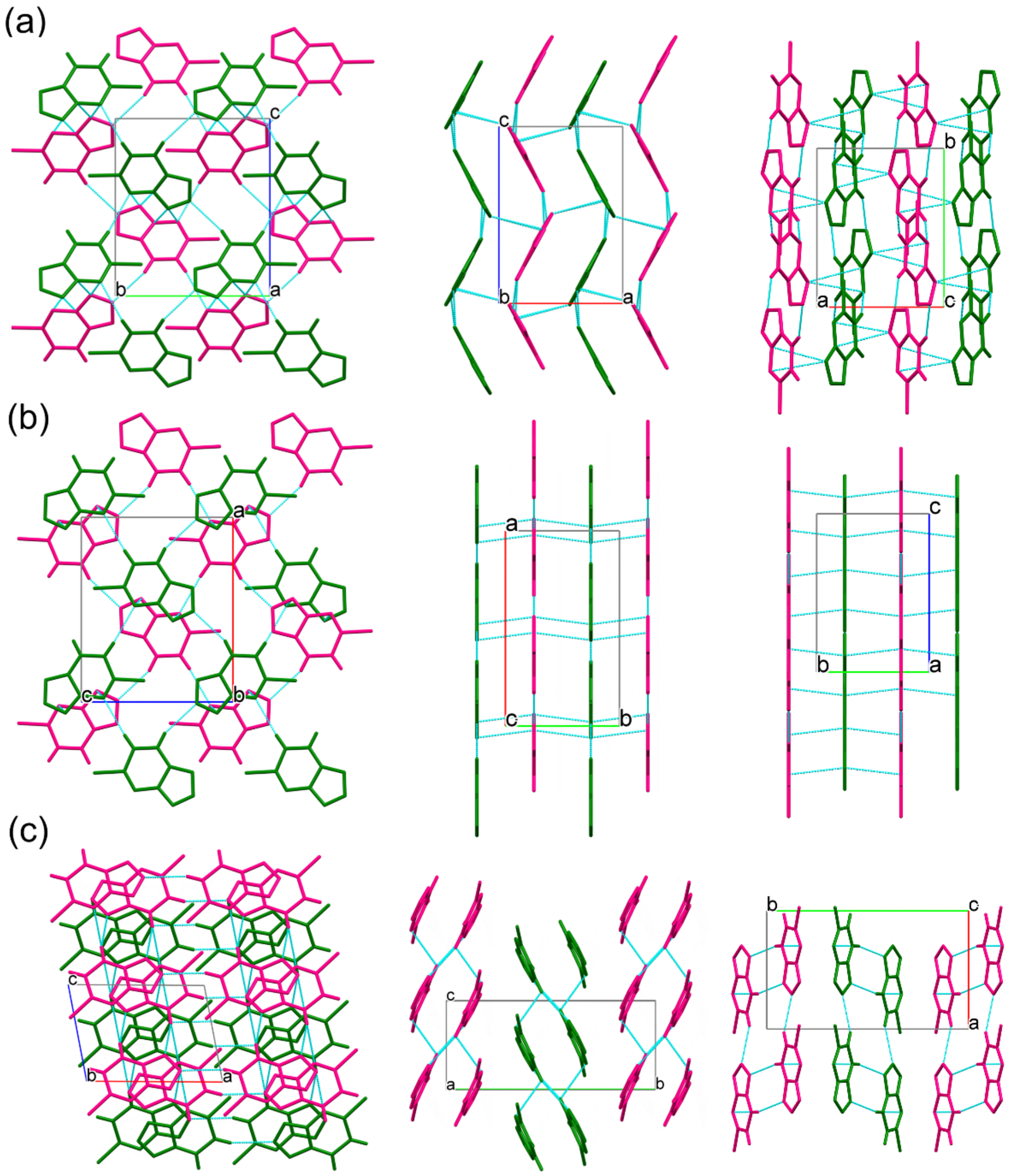

Figure S5 Molecular packing and the short contacts in $C_4H_2N_5Cl$: (a) phase α, (b) α' and (c) phase β. Two layers/double layers indicated by pink and green with the shortest CH···N and N···N interactions are presented.

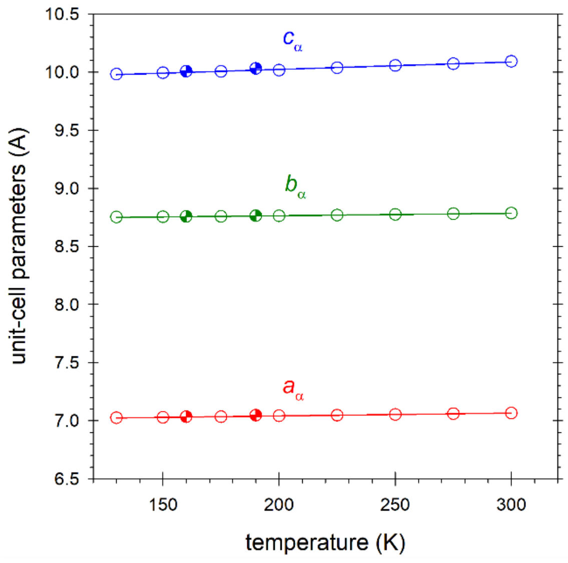

Figure S6 Compression of the unit-cell dimensions in $C_4H_2N_5Cl$ as a function of temperature. The lines joining the points have been drawn for an eye guiding only.

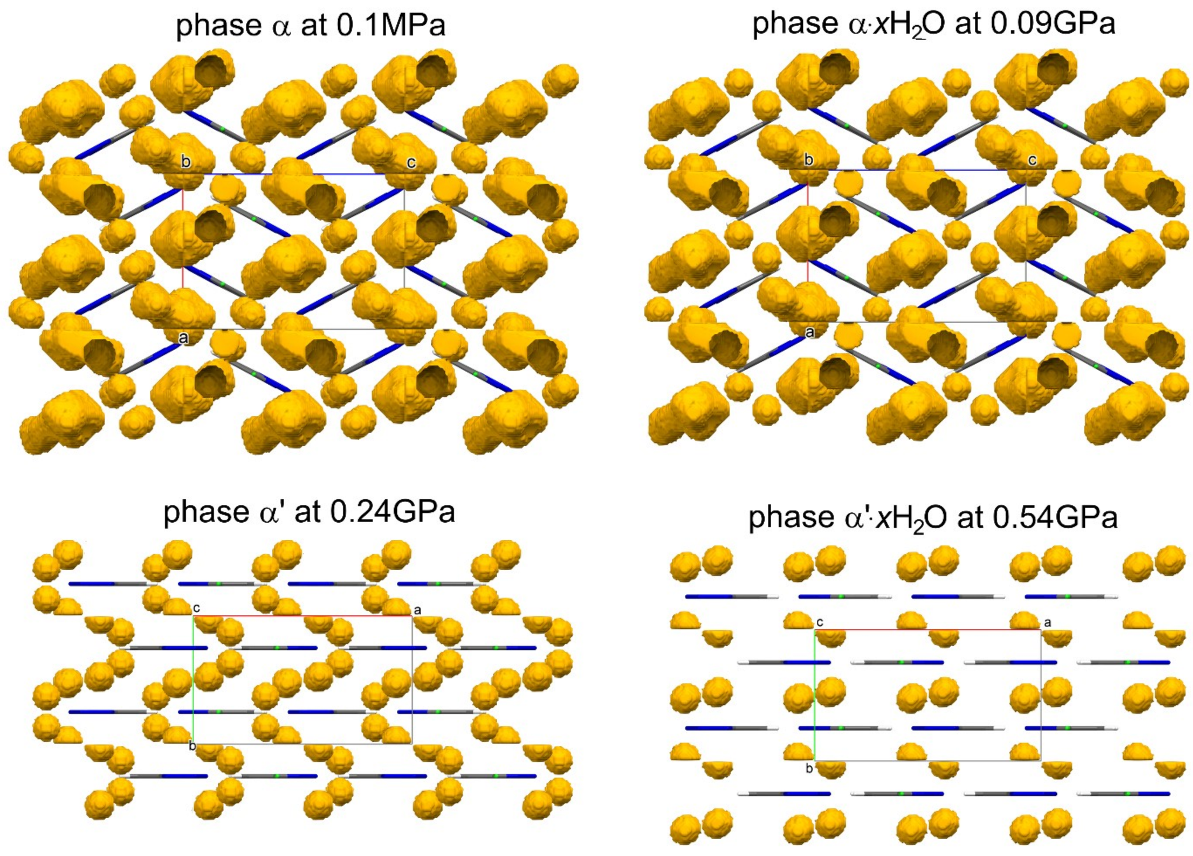

Figure S7 The voids (orange) present in $C_4H_2N_5Cl$ phases: α and α·xH_2O (viewed along [*y*]); α' and α'·xH_2O (viewed along [*z*]), calculated using contact surface with probe radius equal 0.65 Å and grid spacing 0.1 Å.

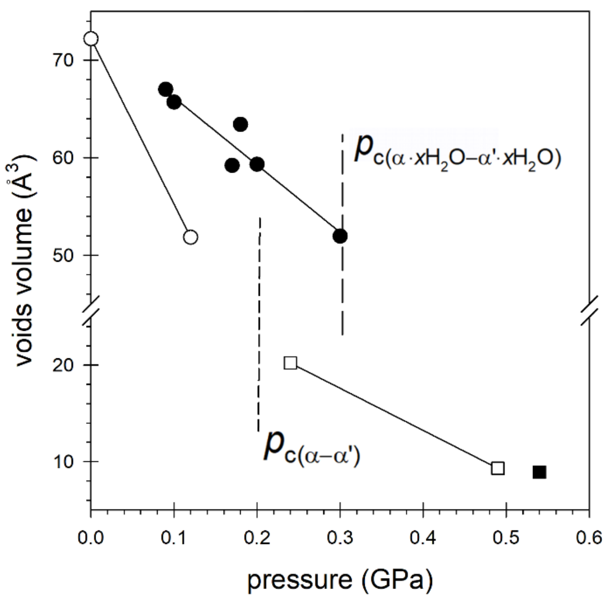

Figure S8 The pressure dependency of voids volume present in phases α (open circles), α·$x$$H_2O$ (full circles), α’ (open squares) and α’·$x$$H_2O$ (full squares), calculated using contact surface with probe radius equal 0.65 Å and grid spacing 0.1 Å.

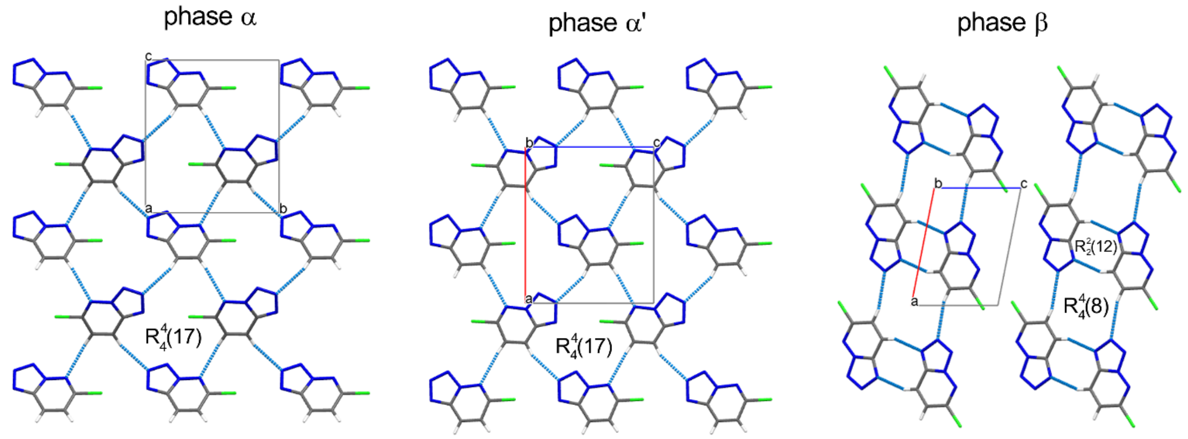

Figure S9 The comparison of hydrogen-bond patterns in $C_4H_2N_5Cl$.

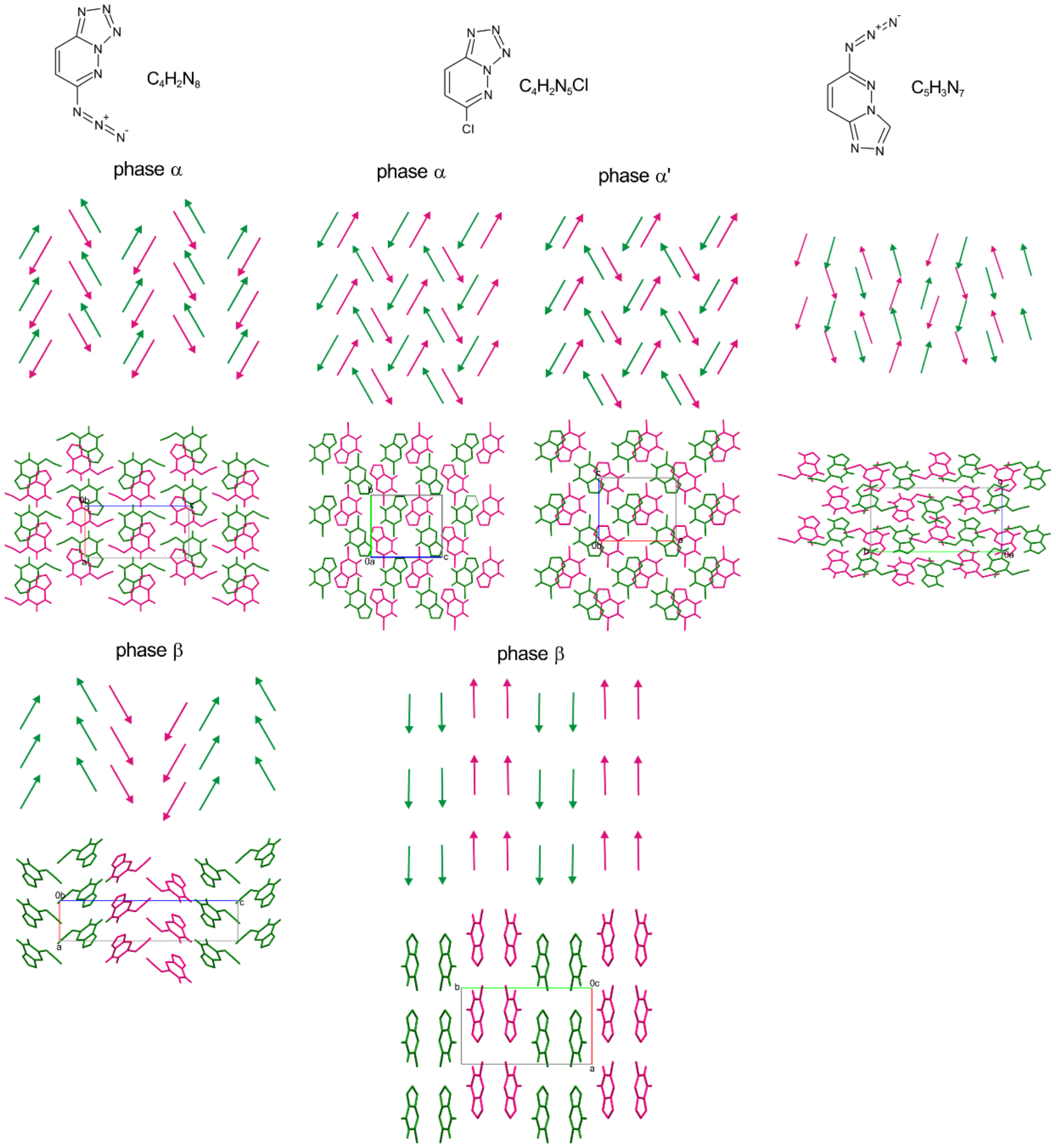

Figure S10 Comparison of molecular packing and the schematic representations of the molecular arrangement as the patterns of arrows in $C_4H_2N_8$, $C_4H_2N_5Cl$ and $C_5H_3N_7$.
